# Rasmussen’s Encephalitis: A Literary Review

**DOI:** 10.7759/cureus.47698

**Published:** 2023-10-26

**Authors:** Abhishek Kumar, Harshil Krishnani, Arundhati Pande, Siddhant Jaiswal, Revat J Meshram

**Affiliations:** 1 Medical Education, Jawaharlal Nehru Medical College, Datta Meghe Institute of Higher Education and Research, Wardha, IND; 2 Paediatrics, Jawaharlal Nehru Medical College, Datta Meghe Institute of Higher Education and Research, Wardha, IND

**Keywords:** nmda receptor-mediated neuronal excitability, epilepsia partialis, hemidisconnection, glur3 antibodies, meningitis, rasmussen's encephalitis

## Abstract

Usually affecting one hemisphere of the brain, Rasmussen's encephalitis (RE) is a persistent inflammatory disease of unclear origin. Rasmussen and colleagues presumed a viral etiology of the sickness in their first description. Later, the condition was linked to autoantibodies that were in the blood. Recently, it was shown that the cause of RE was a cytotoxic T-cell reaction to neurons. RE may be identified histopathologically by cortical inflammation, neuronal degeneration, and cerebral hemispheric-specific gliosis. The hemisphere is affected by increasing multilocular inflammation. To diagnose patients sooner and to evaluate whether the aforementioned phenomena are primary or secondary, it is essential to continue the search for a primary immunological or viral component. This information is crucial for determining the effectiveness of immunotherapy. RE-related seizures can only now be managed surgically. The only procedure that works is complete hemispheric disconnection (hemidisconnection), which may be done as either a (functional) hemispherectomy or hemispherectomy. Although thalidomide has been anecdotally reported, its safety profile prevents it from being used as a first-line treatment despite having a noticeable effect on the frequency and severity of seizures. Finding the disease's root causes more quickly by combining descriptive clinical studies, genetic testing, and early histological evaluation of RE tissue specimens to check for viral and autoimmune pathogenesis. Creating appropriate in vitro or animal models will enable the study of causality, perhaps directing clinical trials.

## Introduction and background

Usually affecting one hemisphere of the brain, Rasmussen's encephalitis (RE) is a persistent inflammatory disease of unclear origin. Rasmussen and colleagues presumed a viral etiology of the sickness in their first description. Monoclonal antibodies in the bloodstream were eventually linked to the illness. However, it has now been shown that a cytotoxic T-cell response toward neurons has a causative role in RE [1]. Serial magnetic resonance imaging (MRI) of RE patients shows that the inflammatory lesion has spread to the affected hemisphere. In a particular brain region, there is a predictable trajectory from increasing volume and T2/FLAIR signal to end stages of atrophy lacking signal abnormalities. Quantitative histopathology measurements suggest that this development may be associated with reduced T cells and reactive astrocytes [2]. These findings support that an initial, active inflammation eventually "burns out." Epilepsia partialis continua (EPC), intractable focal onset seizures, and deterioration of hemispheric functioning are the clinical hallmarks of RE. RE was once thought to be a childhood illness. Adolescent and adult instances were later recorded. Hemispherectomy is the most successful RE therapy in terms of seizure independence. However, this method is often only used on patients who developed persistent hemiparesis and lost the ability to move their fingers with delicacy later in their disease [3]. Several efforts regarding immunotherapy for people suffering from RE have been documented in recent years. These open trials were conducted mainly on individuals not yet impaired enough to be considered candidates for hemispherectomy. These studies show some, though brief, influence on specific illness symptoms. Considering these findings, it is safe to expect that most clinicians who treat RE patients will now consider immunotherapy before hemispherectomy. On the other hand, the lack of information on the long-term history of untreated RE sufferers may make it easier to understand uncontrolled trials in RE [4]. A unique hemispheric ratio approach is used to measure the degree of hemiatrophy in RE in addition to clinical markers (seizure frequency, degree of hemiparesis). This measurement captures the destructive progression of the disease that underlies RE's symptoms and indications [5]. The rationale for conducting this study lies in the imperative to deepen our understanding of this rare and debilitating neurological disorder to develop more effective treatments and improve the quality of life for those affected. The aim of conducting this study is to gain a deeper understanding of its underlying causes, progression, and potential treatment options to improve the quality of life for individuals affected by this rare and debilitating neurological disorder.

## Review

Methodology

Search Strategies

We performed a comprehensive search in the electronic databases PubMed, MEDLINE, Embase, Google Scholar, and ResearchGate and a search of the English-language literature.

Inclusion Criteria

Peer-reviewed journals produced in English, articles published in the last 15 years, the full text of the publication, type of publication: review articles, systematic review, meta-analysis, or empirical studies published in peer-reviewed scientific journals, compliance with the combinations of keywords: clinical features, pathobiology, treatment advances, targeted therapy, and the neurological manifestation.

Exclusion Criteria

Articles published earlier than 15 years, lack of full text of the publication, language of the publication different than English, type of publication different than review articles, systematic review, meta-analysis, or empirical studies published in peer-reviewed scientific journals, malignancy, co-morbidities along with the diseases affecting presentation, specific treatment protocol or than that of the illness in review.

**Figure 1:**
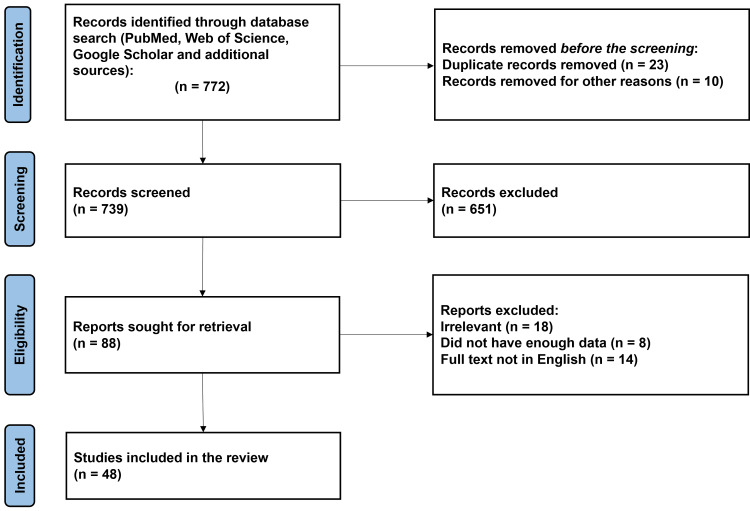
The selection process of articles used in this study. Preferred Reporting Items for Systematic Review and Meta-Analysis (PRISMA) flowchart for the keywords used in the literature review.

Key terms used for the search are "Rasmussen"[All Fields] OR "Rasmussen s"[All Fields] AND "encephalitis"[All Fields] OR "encephalitis"[MeSH Terms] OR "encephalitis"[All Fields]. The Preferred Reporting Items for Systematic Reviews and Meta-Analyses (PRISMA) method used in research methodology is depicted in Figure 1.

Clinical presentation of RE

The progressive illness known as RE is characterized by unihemispheric brain shrinkage, drug-resistant focal epilepsy, progressive hemiplegia, and cognitive deterioration. The condition is unusual and mainly impacts kids and young adults. According to German research, there are 24 occurrences per 10 million children and teenagers countrywide each year [6]. Researchers in the U.K. recently conducted a surveillance study and calculated an incidence of 17 per 10 million children aged 16 and under, comparable to a frequency of 18 per 100,000 people [7]. There has been no mention of gender, geography, or ethnic predominance. The onset age range spans from infancy through adulthood, with an average age of six years. Around eight individuals may have a prodromal phase of moderate hemiparesis with occasional seizures years before the acute event. Seizures from one cerebral hemisphere often occur during the critical period. EPC affects around half of the individuals with RE [8]. As the condition worsens, various focal seizure semiologies indicate newly infected sites of inflammation in the hemisphere. If the language-dominant hemisphere is impacted, children will experience dysphasia, hemianopia, hemiparesis, and cognitive deterioration within a year of the onset of epilepsy [9]. A primary fixed neurological deficiency, motor and mental challenges, and ongoing, challenging-to-treat recurrent seizures describe a generally stable residual stage. RE may manifest in a variety of ways. Around 10% of published clinical instances begin in youth or adulthood [7]. The clinical manifestations are often delayed, and the deficits are much less severe in adults than in children. Additionally, pragmatics may be more typical of temporal lobe epilepsy [10]. Hemidystonia and hemiathetosis are two examples of disease presentations that feature unilateral movement abnormalities. The existence of bilateral illness is debatable. However, it is most likely very uncommon [11]. About two of the roughly half a thousand cases of RE that have been described have histological proof of bilateral disease. The authors are aware of no case of contralateral involvement (even when confirmed by clinical criteria) after unilateral RE was surgically treated. Undiagnosed RE without seizures may contribute to children's unilateral neurological impairments [12]. Patients who have delayed seizure onset or even go up to two years without experiencing a seizure have been shown to have a usually developing RE with typical histology findings. These results suggest that seizures are not a significant side effect of RE [13].

Pathobiology

Cortical inflammation, neuronal loss, and gliosis restricted around one cerebral hemisphere are histological characteristics of RE. Across the hemisphere, inflammation is spreading and multilocular [14]. Pathogenic symptoms include microglial and lymphocytic nodules, perivascular cuffing, neuronal apoptosis, and neuronophagia. End-stage signs include cortical cavitation, astrogliosis, and neuronal cell loss. This result is consistent with an immune-mediated disease comprising T-lymphocyte response, adaptable immunological reactions, and innate immunity supported by microglia and astrocytes [15]. The brain might experience damage in any area. Recent radiological studies, however, confirm long-standing pathological results indicating a propensity for the fronto-insular region, with the occipital cortex being less often afflicted. Patients with brain involvement are often younger and have a more significant illness burden [16]. Indicating a disease process that affects several brain regions at various times is heterogeneity and variety in the lesion location, sickness course, and degree of pathological abnormalities, which are important both within and between people [17].

Antibody-mediated central nervous system degeneration

Although the humoral immune system has traditionally been thought to be unaffected by the brain, recent evidence reveals this is not necessarily true. It has become clear during the last 10 years that several CNS illnesses are linked to potentially dangerous circulating antibodies to neuronal surface proteins [18]. Over the last 10 years, it has become clear that circulating antibodies to neuronal surface proteins are linked to several CNS illnesses that might be dangerous [19]. GluR3 antibodies, on the other hand, were only found in a small number of RE patients who had plasmapheresis treatment [20]. Although this information was not published, Munc-18-1 was found in the blood of a subset of people with RE. Munc-18 is uncertain to be a key target despite being a signaling pathway neuronal protein necessary for synaptic vesicle discharge [7]. Munc-18 is an intracellular protein necessary for synaptic vesicle discharge and is an evolutionarily conserved neuronal protein; nonetheless, it is uncertain to be a big focus [20].

T-cell cytotoxicity

The role of T lymphocytes in the genesis of RE is crucial. Around 10% of inflammatory T cells are CD8-positive and contain granzyme B. Granzyme B cells containing cytotoxic granules directed toward the target cell membrane were found close to neurons as well as astrocytes; granzyme B release onto neurons was seldom observed [21]. In addition, spectral analysis of T cells of brain lesions indicated that these cells originated from various antigenic and epitope-responding progenitor T cells, demonstrating the specificity of single-brain antigens [22]. Finding these driving antigens in the brains of RE patients is the subsequent step. Because cytotoxic T lymphocytes assault neurons and astrocytes, both cells were expected to express an autoantigen [23]. The antigen's identity, however, is yet unknown. Another hypothesis is that these cytotoxic T cells identify foreign antigens from pathogen-infected neurons and astrocytes rather than autoantigens. Pathogen-infected cells in a specific area of the brain may explain the disease's unilateral predominance. In the past, several viruses including Epstein-Barr virus, Cytomegalovirus, herpes simplex, and enterovirus were examined in the brain tissue of RE patients [24]. 

Inflammatory gene expression

In 12 samples of RE brain tissue, to find out the nature of the immune reaction under this condition, researchers compared the relative expression levels of 86 mRNA transcripts connected to inflammation with autoimmune. They were compared to samples taken from a group of 12 people who had cortical dysplasia. Individuals having cortical dysplasia have localized seizures, defects in cortical neuron growth, and histological signs of the inflammatory reaction, but less severe than RE. The research discovered that the RE cohort had higher expression levels of a subset of seven functionally important genes, including interferon-, CCL5, CCL22, CCL23, CXCL9, CXCL10, and Fas ligand, than did the cortical dysplasia cohort. Such genes often include those that encode specific chemokines connected to the activation of helper and inducer T cells and memory and effector T cells, which might also help draw these T cells to the site of inoculation [25]. The problem is that none of the above-mentioned trials were carried out before the disease started. Therefore, it is necessary to clarify the main pathophysiology. Many discoveries could not be primary but after the sickness process. To diagnose patients sooner and to evaluate whether the phenomena are primary or secondary, it is essential to continue the search for a primary immunological or viral component. This information is crucial for evaluating the effectiveness of immunotherapy [25].

Microglia-induced degeneration

One of the neuropathological features of RE is microglial activation [1]. These cells' levels of activation vary depending on where in the brain they are, but they closely coincide with the stages of cortical damage progression and T-cell infiltration pattern [26]. Microglia produce seizures in epileptic conditions by releasing interleukin and other pro-inflammatory cytokines. Additionally, synaptic stripping caused by complement may be carried out by activated microglia, increasing network excitability [27]. It is still being determined exactly how microglial cells contribute to RE's pathogenesis. Along with microglia, astrocytes are activated in RE. The course of cortical injury is strongly resembled by the pattern of astroglia activation [15,23]. Numerous epileptic and inflammatory brain diseases may be caused by astrocytes [28]. As a result, astrocytes are likely to have a comparable function in the immune response in RE. However, astrocytes are destroyed as the illness progresses, most likely due to a cytotoxic T-cell response. CSF albumin concentration rises with illness progression in RE [29]. Inward-rectifying potassium (Kir 4.1) channels in astrocytes are downregulated after albumin absorption, which reduces the buffering of extracellular potassium and NMDA receptor-mediated neuronal excitability. As a result, albumin leakage through astrocyte regulation may promote seizure start during RE [25].

Treatment of seizures

Antiepileptic drugs' impact on seizures and the onset of sickness in RE is minimal. Epilepsy partialis continua is resistant to antiepileptic drugs. Seizure medicine treatment should focus on protecting the patient from the most severe seizures, particularly bilateral convulsive seizures, rather than trying to achieve seizure independence. To get the greatest seizure control with the fewest side effects, medication must be customized [30]. Injecting botulinum toxin into the zygomaticus for the face myoclonus and into the muscles of the upper limb for localized EPC significantly reduced unpleasant spasms of the face and enhanced functional use of the limb [31]. A case series of brief intensive immunotherapies was presented, including a case report and open-label study employing rituximab infusions. These interventions included steroid pulses with apheresis therapy [32]. Medicines have demonstrated a favorable short-term impact on seizure frequency. 2011 saw case reports of transcranial magnetic stimulation and vagus nerve stimulation used to treat seizures. However, most people with RE resist antiepileptic drugs [33].

Surgery

Currently, the only treatment for seizures brought about in RE is surgery. Since entire hemispheric disconnection (hemidisconnection), also known as (functional) hemispherectomy or hemispherectomy, is the only procedure that works, this seems to have practical ramifications [33]. Homonymous hemianopia and hemiplegia are inevitable, even if they may both already exist. While unassisted walking is a goal of therapy, fine motor activity in the afflicted hand is still not. Small resections may be preferred to maintain function in some instances. However, no researchers have observed persistent seizure freedom following restricted resection in RE patients. For individuals with RE, hemispherectomy has one of the highest long-term seizure-free success rates (>70-80% long-term seizure-free result) [34]. If the illness impacts the dominant hemisphere, older individuals may find the decision much more challenging [34].

From pathogenesis to treatment

RE Immune Agents

**Figure 2:**
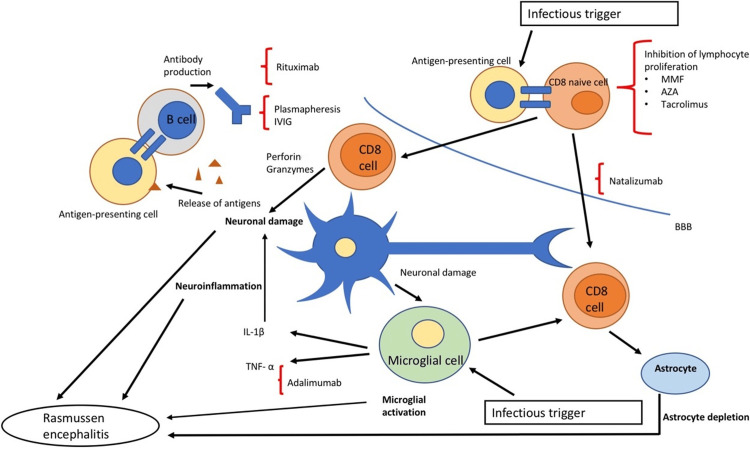
Pathogenetic model of Rasmussen encephalitis. This image is taken from an open-access journal licensed under a Creative Commons Attribution 4.0 International License (Orsini et al. [40]). MMF: Mycophenolate mofetil

Many immune-stimulating medications have been suggested to treat RE patients based on that pathophysiology. In the literature, there are only a few well-planned clinical studies on the immunological approach to RE, mainly including corticosteroids and intravenous immunoglobulin (IVIG); however, more data use other immunological agents [35]. In the past, corticosteroids have been recommended as first-line treatment in patients with RE during the beginning of the disease and exacerbations with variable posology. Most studies showed that methylprednisolone taken at high doses was effective [36]. Innate and adaptive immunological responses, as well as transcriptional and posttranscriptional mechanisms, are affected by corticosteroids. Their therapy affects protein synthesis, the stability of other genes' mRNA, and the transcription of certain pro-inflammatory genes, mainly IL-1, IL-2, IL-6, TNF alpha, and adhesion molecules [37,38]. However, this broad-spectrum activity has significant adverse effects, primarily on youngsters. Corticosteroids are thus usually not recommended for long-term maintenance treatment and are only used in the acute stage of the disease. Most scientists concur that IVIG has an immunomodulatory and anti-inflammatory impact that may be utilized to treat several autoimmune illnesses, mainly affecting the nervous system (such as Guillain-Barré syndrome and others) [39]. The pathogenetic model of RE is elicited in Figure 2.

**Figure 3:**
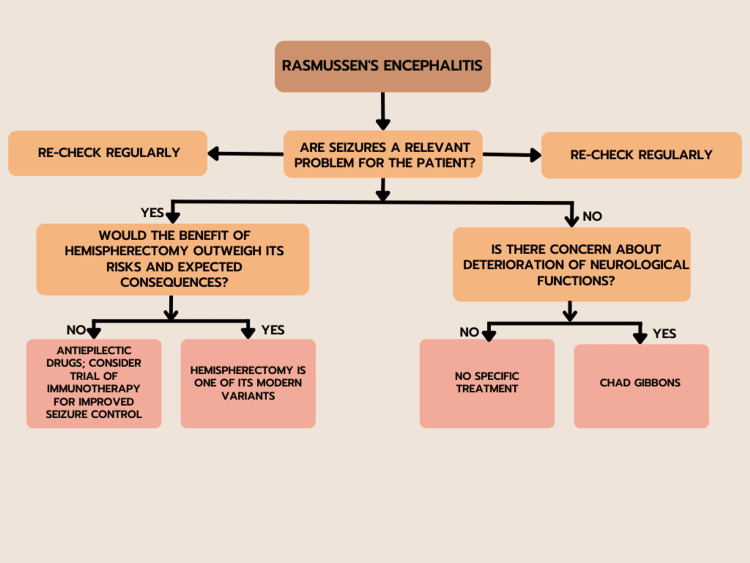
Therapeutic approach to Rasmussen encephalitis.

In addition to T-induced damage unrelated to the etiology of RE, IVIG also affects antibody-mediated damage by decreasing the number of costimulatory pathways [41]. The combination of IVIG plus corticosteroids, often administered to RE patients, has been shown to provide only partial or momentary effects. On the other hand, it was discovered that giving two adult patients with RE high-dose IVIG in the acute phase, followed by maintenance therapy, led to a delayed but lasting response [42]. The use of plasmapheresis in RE patients has already been described in various clinical trials; however, the procedure's effectiveness is uncertain, and the response is typically transient. Plasmapheresis lowers antibody titer while boosting the clearance of other immune mediators and complement fractions [43]. Most likely as a result of a lack of response to T-induced cellular injury. Of all immunosuppressive medications, tacrolimus has the most research supporting its use in treating RE. The medicine has been suggested as a stand-alone treatment and switch therapy after corticosteroid usage. It affects the T cell immune response by preventing the generation of IL-2. Patients receiving tacrolimus showed a slowing of the illness without having a substantial effect on seizures in research published by Lagarde et al. [44]. Tacrolimus was linked to a similar clinical response to IVIG treatment but a higher risk of side effects. The use of additional immunosuppressive medications has sometimes been documented. The purine synthesis inhibitor azathioprine (AZA) was used in several cases which improved seizure frequency but had no protective effects on the progression of cognitive loss. Mycophenolate mofetil (MMF), has been successfully administered in several juvenile cases of autoimmune encephalitis [45]. It blocks purine production in lymphocytes, causes apoptosis in activated T cells, and lowers lymphocyte recruitment. Since T cells are crucial to the pathophysiology of RE, this medication may target them. It has only been used a few times to treat RE patients, but it has been successful in managing the condition both alone and in combination with steroids. Furthermore, MMF showed fewer side effects than AZA. Thalidomide has been reported anecdotally, but despite a discernible impact on the frequency and severity of seizures, the drug's safety profile precludes its use as a first-line therapy [46,47]. The therapeutic approach to RE is elicited in Figure 3, and medications acting on the immune system are used in RE, described in Table 1.

**Table 1:** Medications acting on the immune system are used in Rasmussen encephalitis. Drugs acting on the immune system are used in Rasmussen encephalitis [5,6,9,30,34,36,38,43,46]. IVIG: Intravenous immunoglobulin; BBB: blood–brain barrier

Drug	Mechanism of action	Clinical implications
Corticosteroids (high-dose methylprednisolone)	The broad-spectrum post-transcriptional and transcriptional anti-inflammatory action. Modulation of the expression of adhesion molecules such as cytokines.	Often switching between IVIG and other immunosuppressive drugs. Effective during the short term.
Intravenous immunoglobulin	Reduction of harm caused by antibodies. Immune control through several antibody-free routes (cytokine modulation, complement downregulation).	Often in conjunction with corticosteroids. Varying effectiveness, ranging from short-term, partial responses to long-term responses.
Plasmapheresis	Reduction in the amount of soluble immunological mediators and circulating antibodies.	Partial and fleeting reaction. unknown long-term advantages
Tacrolimus	Calcineurin blocker reduces IL-2 synthesis, essential for T-cell activation and proliferation.	It is effective in delaying the onset of illness but ineffective in managing seizures. More serious adverse events than with IVIG.
Azathioprine	Creating a purine analog as a purine synthesis inhibitor (6-mercaptopurine) reduces lymphocyte clonal growth.	It is effective in reducing seizures but ineffective in delaying or stopping disease development.
Mycophenolate mofetil	Inhibitor of inosine monophosphate dehydrogenase, which inhibits the production of purines, decreases lymphocyte recruitment and proliferation, and causes the death of activated T lymphocytes.	Promising outcomes in terms of safety and effectiveness.
Thalidomide	Inhibition of innate and adaptive immune responses through a variety of ways. Indirect inhibition of NF-.	Not a viable first-line choice.
Rituximab	Chimeric anti-CD20 monoclonal antibody. Targets B cells, inhibiting their proliferation and reducing antibody production.	Partial effectiveness in halting disease development and symptom progression. Excellent safety profile.
Adalimumab	A monoclonal anti-TNF antibody with humanization.	Effective against seizures; ineffective against cognitive deterioration.
Natalizumab	Antibody against the alpha 4 integrin that has been humanized reduces T cells' capacity to traverse the BBB.	Promising outcomes in terms of safety and effectiveness.

Results and discussion

There are still several difficulties in providing clinical treatment for RE patients. Functional hemispherectomy remains the only clear treatment option despite advances in our knowledge of the immunological causes of the illness. When it comes to delaying the development of sickness and lowering the frequency and severity of clinical signs like seizures, medical therapy is often ineffectual [48]. Patients with sluggish disease progression or those ineligible for surgery benefit greatly from a pharmaceutical strategy using medications that impact the immune system, especially T cellular immunity. More T-targeted medications will hopefully be developed in the next years to provide people with rapidly progressing diseases an option for surgery, enhancing clinical outcomes and long-term quality of life for RE patients [34]. Despite the lack of biochemical disease biomarkers or their recent exploratory study, neuroimaging has emerged as a possible disease biomarker. Finding anything especially connected to RE and creating more accessible biomarkers may be accomplished using label-free quantitative proteome analysis of CSF and nano-high-performance liquid chromatography with electrospray-ionization quadrupole time-of-flight mass spectrometry. Pathobiology and medications used for other autoinflammatory disorders of the grey matter, such as early-stage multiple sclerosis, may be useful for treating RE [46,48].

Some decrease the course of diseases, but none have completely healed or even stopped them. Immunotherapy has changed the course of the disease, favoring more prolonged and milder hemibiotrophic and hemiplegic episodes. This change may influence surgical choices, although it is unknown if it has had a major effect on the course of the illness. There are more and more instances when immunotherapy has slowed the patient's functional deterioration but hasn't stopped the patient from having frequent, incapacitating seizures, which they may have had for years [25,41]. A therapeutic conundrum results from such a circumstance; hemispherectomy is not advised because of the unavoidable functional impairments that will follow surgery. However, there is a genuine danger that therapies to slow down disease development may push back the timing of the ultimate surgical procedure beyond the point at which the best possible post-hemispherectomy results can be anticipated. Immunotherapies and the accompanying viral or antibody biomarkers in patients with acute encephalitis have altered clinical practices and patient outcomes [15,36,42,44,48].

## Conclusions

Clinical treatment for RE remains challenging. Functional hemispherectomy is the primary treatment despite advances in understanding immunological causes. Medical therapy often fails to delay the disease or reduce seizures. Some benefits from immune-targeting medications and more may be developed. Neuroimaging is a potential biomarker. Label-free proteome analysis and chromatography-mass spectrometry can help create accessible biomarkers. Medications for similar gray matter disorders may help treat RE. Due to the rarity of RE, urgently needed global collaboration studies are needed for the prospective collection of archival samples and enrollment in treatment trials. Forecasting how treatments will advance until the causes of RE are discovered is difficult. Over the last ten years, a number of immunotherapy programs have been launched. Immunotherapy has altered the disease course but doesn't always stop frequent seizures. Delaying surgery for immunotherapy may affect outcomes. By combining descriptive clinical investigations, genetic testing, and early histological screening of RE tissue specimens to look for both viral and autoimmune pathology, research efforts to find the disease's underlying causes should be expedited. The investigation of cause and effect will be made possible by the development of suitable in vitro or animal models, perhaps guiding clinical trials. Suitable models may guide clinical trials.
